# A Flexible Integrated Bending Strain and Pressure Sensor System for Motion Monitoring

**DOI:** 10.3390/s21123969

**Published:** 2021-06-09

**Authors:** Rou Feng, Yifeng Mu, Xiangwen Zeng, Weijie Jia, Yuxuan Liu, Xijun Jiang, Qibei Gong, Youfan Hu

**Affiliations:** 1 Hunan Institute of Advanced Sensing and Information Technology, Xiangtan University, Xiangtan 411105, China; 201821521392@smail.xtu.edu.cn (R.F.); 201721521305@smail.xtu.edu.cn (Y.M.); 202021001531@smail.xtu.edu.cn (Y.L.); 202021001515@smail.xtu.edu.cn (X.J.); gqb_taoking@smail.xtu.edu.cn (Q.G.); 2 Key Laboratory for the Physics and Chemistry of Nanodevices and Center for Carbon-Based Electronics, Department of Electronics, Peking University, Beijing 100871, China; zengxiangwen@pku.edu.cn (X.Z.); jiaweijie@pku.edu.cn (W.J.)

**Keywords:** flexible sensor, integrated sensor system, composite structure, bending strain, pressure

## Abstract

Flexible sensors have attracted increasing research interest due to their broad application potential in the fields of human–computer interaction, medical care, sports monitoring, etc. Constructing an integrated sensor system with high performance and being capable of discriminating different stimuli remains a challenge. Here, we proposed a flexible integrated sensor system for motion monitoring that can measure bending strain and pressure independently with a low-cost and simple fabrication process. The resistive bending strain sensor in the system is fabricated by sintering polyimide (PI), demonstrating a gauge factor of 9.54 and good mechanical stability, while the resistive pressure sensor is constructed based on a composite structure of silver nanowires (AgNWs) and polydimethylsiloxane (PDMS)-expandable microspheres with a tunable sensitivity and working range. Action recognition is demonstrated by attaching the flexible integrated sensor system on the wrist with independent strain and pressure information recorded from corresponding sensors. It shows a great application potential in motion monitoring and intelligent human–machine interfaces.

## 1. Introduction

Recently, flexible sensor systems have been widely explored with applications in intelligent robotics [1,2,3,4], electronic skin [5,6,7,8], wearable electronic [9,10,11], etc., because of their superiority in portability and conformability for a better human–computer interface. In the past few years, it has been well demonstrated that physiological information of the human body, such as pulse [12,13], blood pressure [14], body temperature [15,16], electrocardiogram [17], etc., and the mechanical status of the human body, including movements and postures [18,19], can be monitored by flexible sensor systems when they are properly attached to different areas on the human body, providing valuable data for health and sports status assessments and clinical diagnosis. Currently, one challenge faced by the flexible sensor systems is that the manufacturing process is complex, which makes the integration more difficult than that of rigid sensors, and the production cost is high. Meanwhile, as the system becomes more complex to involve different sensors for multiple information capture, the ability to discriminate different stimuli is highly desired.

In this work, we report a flexible integrated sensor system that was constructed via a feasible low-cost fabrication approach. The integrated system consists of two functional modules, a bending strain sensor which is based on graphite wires with a serpentine structure that sintered from polyimide (PI), and a pressure sensor which is based on a composite structure of polydimethylsiloxane (PDMS)-expandable microspheres and silver nanowires (AgNWs). The bending strain sensor shows good sensitivity and excellent cycling stability, while the pressure sensor possesses a tunable sensitivity and working range. In the integrated system, these two sensors can respond to strain and pressure independently without crosstalk. Discrimination of external stimuli by the system for motion monitoring on the wrist is demonstrated, which reveals its great application potential in the field of sports monitoring and wearable electronic devices.

## 2. System Structure and Device Fabrication Method

### 2.1. System Structure

**Figure 1:**
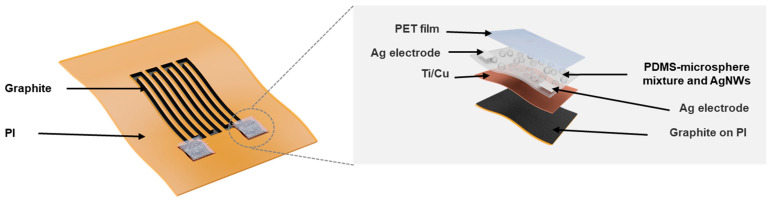
Structure diagram of a flexible integrated bending strain and pressure sensor system.

Figure 1 shows a schematic diagram of a flexible integrated bending strain and pressure sensor system. The strain sensor is a graphite serpentine wire patterned by laser sintering on a PI tape, and its two ends are deposited with squared Ti/Cu (20 nm/50 nm) metal films as two electrodes. There are two pressure sensors integrated into the system, which are constructed on the top of the strain sensor’s two electrodes. As enlarged in the right part of Figure 1, the pressure sensor’s key sensing component is a layer of PDMS-expandable microsphere mixture coated with AgNWs. In addition, there are two Ag pads added on the top surface of the layer at both sides to serve as the electrodes for the pressure sensor, and the top of the pressure sensor is covered by a PET film.

### 2.2. Device Fabrication

**Figure 2:**
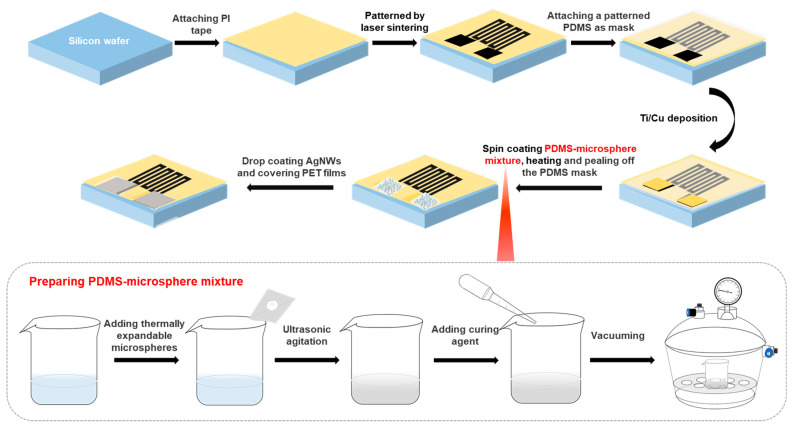
Schematic diagram of the fabrication process of the integrated system. The part in the grey dotted box at the bottom is the preparation process of the PDMS-microsphere mixture.

Figure 2 shows a schematic illustration of the fabrication procedures of the integrated system. A silicon wafer was used as the supporting substrate during the fabrication process, which was purchased from Tianjin SEMI Tech. Res. Inst. First, the silicon wafer was cleaned by rinsing in deionized water, acetone, and isopropanol in sequence, and then a 50 μm-thick commercial PI tape was attached to the silicon wafer. Laser-induced porous graphite was patterned on the PI film with a serpentine structure by the direct writing of a CO_2_ laser [20] (Universal laser systems, PLS 6MW). After attaching a piece of patterned PDMS membrane as a mask on the substrate, a Ti/Cu metal film with a thickness of 20 nm/50 nm was deposited on the two ends of the serpentine graphite wire as electrodes by using DC sputtering (PVD75, Kurt J. Lesker). Then, the PDMS-expandable microsphere mixture was spin-coated on the substrate and heated on a hot plate at 150 °C for 3 min to cause the embedded microspheres to expand and protrude from the PDMS surface to form microstructures [21]. Then, AgNWs dispersed in ethanol solution were dropped onto the surface of the PDMS-expandable microsphere structure and continued to be heated for another 2 min. Finally, the PDMS mask was removed, and two polyethylene terephthalate (PET) films with an area of 1 × 1 cm^2^ were put on the top of the pressure sensors after two Ag electrodes were added on the surface by using Ag pastes (PELCO^®^ Conductive Liquid Silver Paint) to accomplish the sensor fabrication.

The part in the grey dotted box of Figure 2 shows the process to prepare the PDMS-microsphere mixture. First, Expancel 043 DU08 thermal expandable microspheres were added into the prepolymer of PDMS (Sylgard 184, Dow Corning) at a weight ratio of 0.4%, and ultrasonic agitation was carried out for 2 h to make microspheres dispersed homogeneously in the prepolymer. Then, the curing agent of PDMS was added into the prepolymer at a weight ratio of 10:1, and the mixture was pumped in a vacuum tank until there was no bubbling in the mixture.

## 3. Results and Discussions

### 3.1. Characterization of Strain Sensor

**Figure 3:**
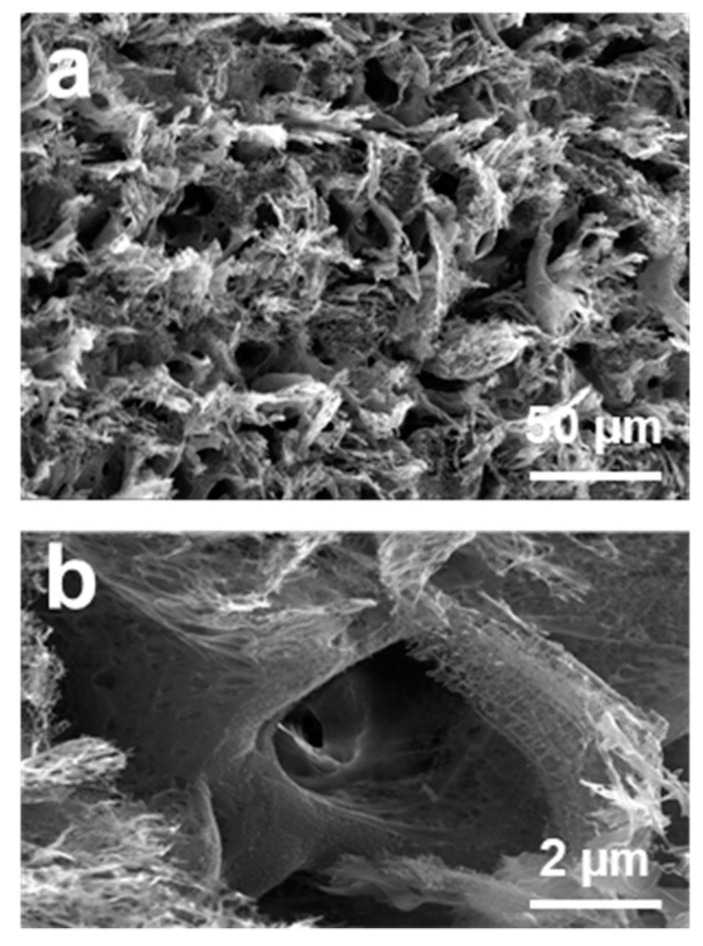
(**a**,**b**) SEM images at different magnifications of the sintered PI tape surface, showing porous graphite microstructures.

**Figure 4:**
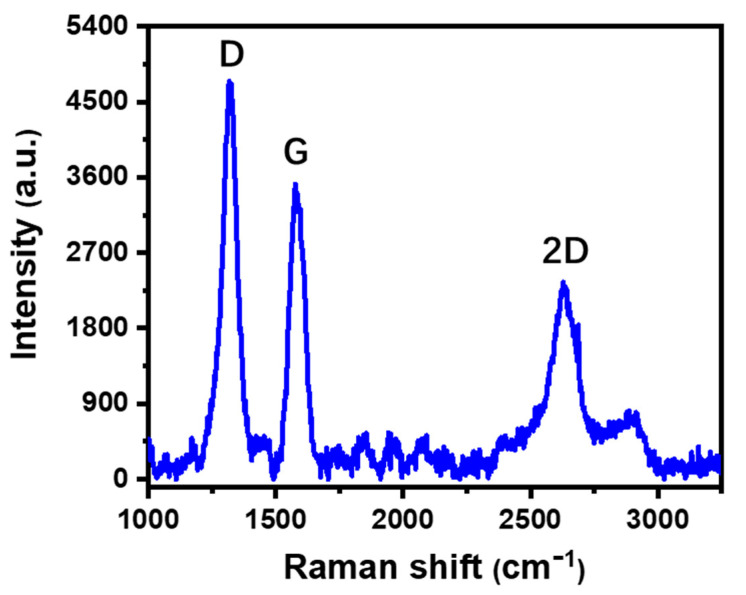
Raman spectrum of sintered PI tape.

We first checked the morphology of the strain sensor. From the scanning electron microscope (SEM) images shown in Figure 3, it is clear that there were porous graphite microstructures on the surface of PI film, and the carbonized structure contained multilayer graphene flakes [22]. The carbonization process of the PI film under laser irradiation was most likely by a photothermal mechanism [23,24], during which the PI film absorbed the incident laser energy and converted it into heat to induce extremely high temperature in the irradiated region to result in carbonization of the film [25]. Some chemical bonds in the PI film, such as the C-O, C=O and N-C bonds, could be easily broken under this high temperature [24]. To further confirm the microstructure, Raman spectrum measurement was carried out on the sintered PI film, as shown in Figure 4. It shows three prominent peaks: the D peak around 1350 cm^−^^1^, the G peak around 1580 cm^−^^1^, and the 2D peak around 2700 cm^−^^1^. The spectrum was similar to that of porous graphite microstructures obtained in a previous report [22].

**Figure 5:**
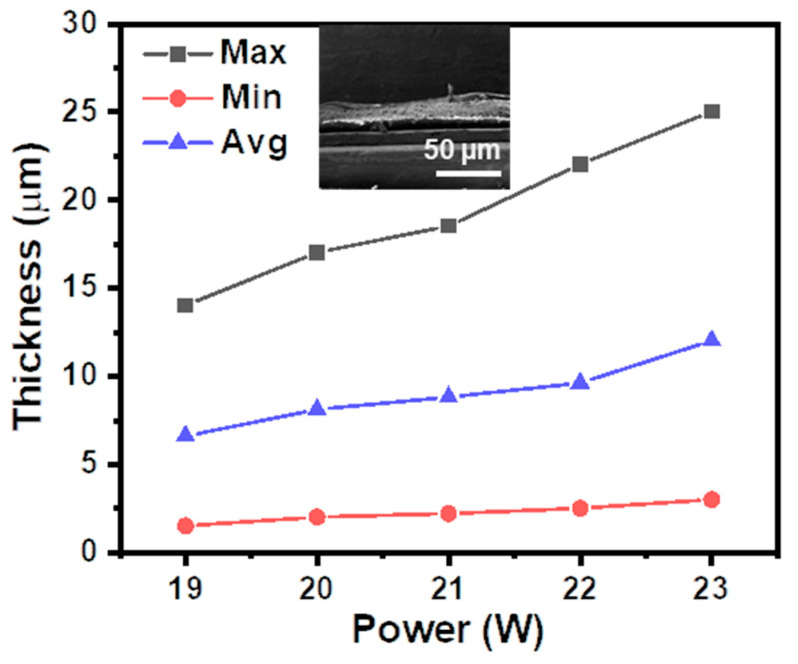
Effect of the laser power on the thickness of the obtained graphite on PI tape surface. Inset: cross-section SEM image of the obtained graphite on PI surface.

The power of the laser during sintering is one of the most important parameters in the process that need to be controlled to introduce desired porous graphite structures without excessive ablation of the underlying thin PI film to make it fragile. The thickness of laser-sintered graphite on the PI surface at different laser powers was estimated by a step profiler (Ambios, XP1), which is shown in Figure 5. As indicated by the inset cross-section SEM image, the obtained porous graphite structure was not even and had a waved surface on the PI film. So, the thickness at the maximum and the minimum were both recorded, and the calculated average thickness of the obtained graphite showed a linear relationship with the laser power. When the laser power increased from 19 W to 23 W, the maximum thickness of the graphite layer increased from less than 15 μm to above 25 μm. We conducted mechanical deformation tests for all the obtained graphite sintered at different laser power conditions. It was found that when the laser power was high, the graphite structure would be partially exfoliated from the PI substrates after several cycles of deformation. Meanwhile, when the laser power was low, the fluctuation of the obtained graphite’s resistance was relatively large. So, in the following experiments, a laser power of 21 W was used to ensure a high mechanical robustness and electrical reproducibility of the obtained strain sensor.

**Figure 6:**
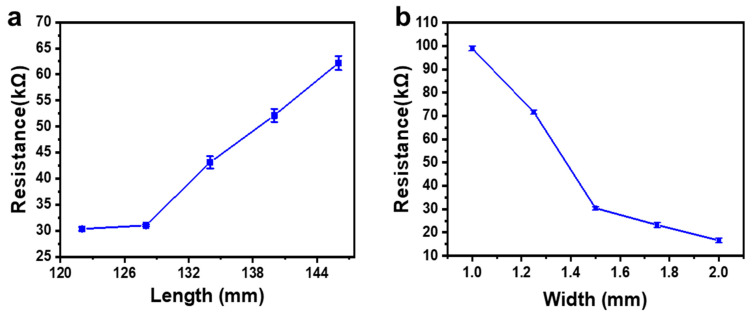
Change in resistance with (**a**) the length and (**b**) the width of the wires in the serpentine structure.

We also checked the main parameters affecting the resistance of the obtained strain sensor, including the length and width of the wires in the serpentine structure. The results are shown in Figure 6a,b, respectively, in which every data point was obtained by average the resistances measured from three identical devices fabricated with the same conditions. As for resistance, *R*, *R = ρL/S*, where *ρ* is the resistivity, *L* is the length, and *S* is the cross-sectional area of the wire. The results showed that the resistance of the laser-sintered graphite was proportional to the length of the wire, while inversely proportional to the width of the wire. It should be mentioned that it was not a strict linear relationship as predicted by the above equation. This may come from the scattering-induced interaction area extension at the wire edge during sintering and the uneven thickness of the obtained graphite. Even so, the controllability of the resistance over the geometric dimension was desirable based on this feasible “laser writing” approach which had great flexibility for device design.

**Figure 7:**
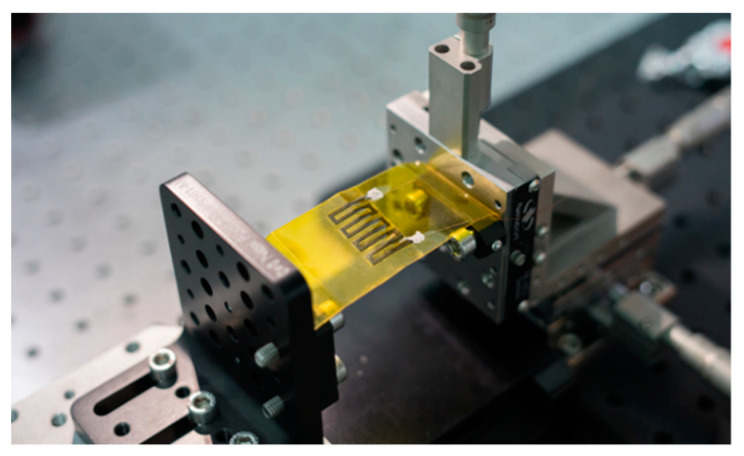
Optical image of the experimental setup for bending strain test.

The experimental setup for the bending strain test is shown in Figure 7, in which the PI substrate was captured between two sample stages mounted on an optical table. One stage (Newport, 9101NF) was fixed, and the other one (Newport, M-462-XYZ-M) was moveable to introduce different bending strains in the PI substrate and thus the strain sensor.

**Figure 8:**
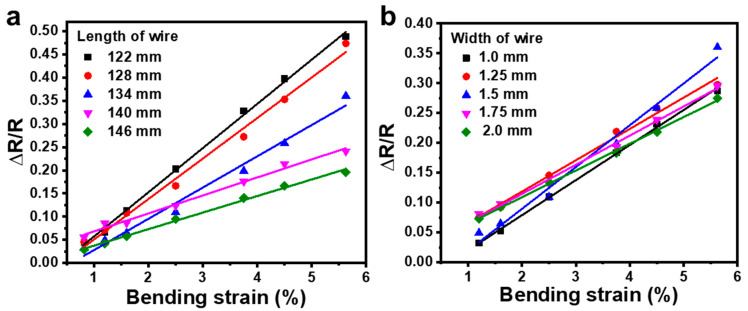
Change in relative resistance with bending strain when the devices were constructed with (**a**) different lengths and (**b**) different widths of wires in the serpentine structure.

Figure 8a,b shows the relative resistance change, Δ*R/R*, of the strain sensor with different geometries in response to different bending strains, ε, where Δ*R* and *R* are the variation of resistance and the initial resistance of the sensor without strain. The calculated gauge factor, $GF=\frac{\delta \left(\frac{\Delta R}{R}\right)}{\delta \varepsilon }$ of these devices decreased from 9.54 to 3.5 as the length of the wire increased from 122 mm to 146 mm, while it showed no obvious dependence on the width of the wire when it doubled from 1.0 mm to 2.0 mm. This different dependence on the two geometry factors comes from the working mechanism of the sensor, which is based on the resistance change induced by the relative displacement of flakes in the graphite under deformation [26]. As referring to Figure 4, when the device was bent, most deformation was introduced along the length direction, and thus the GF of the sensor was significantly influenced by the wire length, while the width of the wire had a negligible effect on the response of the device. When the length of the wire increased, the resistance increased more quickly than the change in resistance variation under deformation, resulting in a decreased GF with increased wire length.

**Figure 9:**
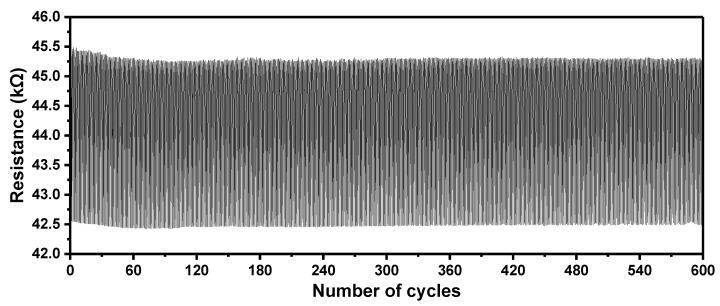
Mechanical stability test of the strain sensor under 600 loading-unloading cycles of a 5.6% bending strain.

We further tested the stability of our strain sensor by performing 600 cycles of loading-unloading of a 5.6% bending strain. The result is shown in Figure 9. The resistance fluctuating of the strain sensor was less than 1% during these 600 cycles, revealing excellent mechanical robustness.

### 3.2. Characterization of the Pressure Sensor

**Figure 10:**
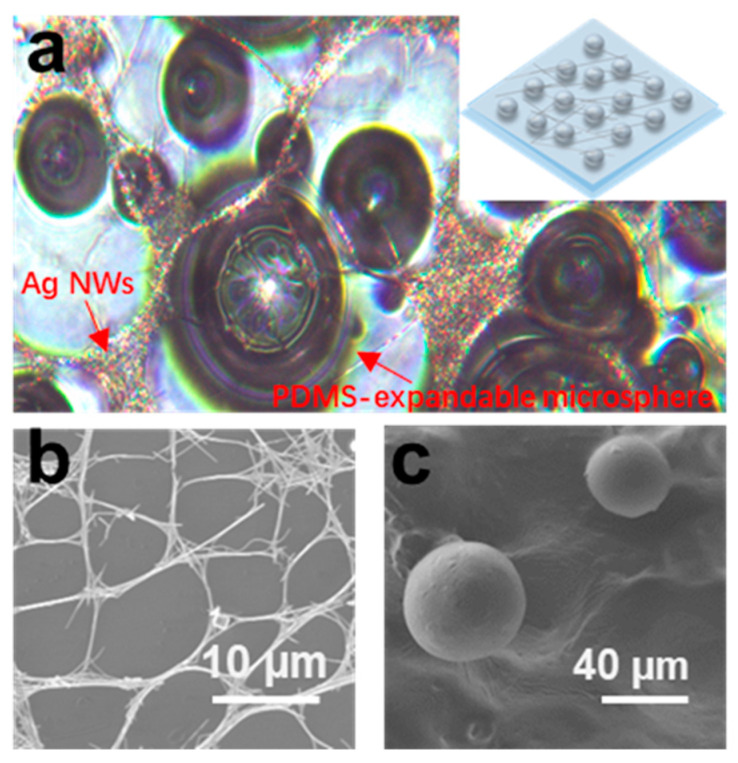
(**a**) Optical image of PDMS-expandable microsphere structure covered with AgNWs. The inset: Schematic image of the structure. SEM image of (**b**) the distribution of AgNWs and (**c**) the morphology of microspheres after expansion.

The optical image in Figure 10a shows the surface morphology of the sensitive layer in the pressure sensor. It presents that there were protruded microspheres on the PDMS surface and they were partially covered by AgNWs. The SEM images in Figure 10b,c show that AgNWs were distributed in a network form with a lot of holes on the surface, and the diameters of the microspheres embedded in the PDMS layer after expansion were around 50–70 μm. When the pressure sensor was pressed, the resistance between two Ag electrodes would be changed due to the change in the conduction paths that were modified by different deformations of the AgNWs networks.

**Figure 11:**
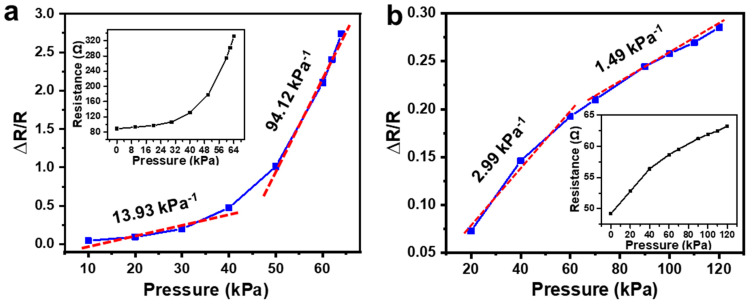
Pressure response of pressure sensors fabricated with a AgNWs solution concentration of (**a**) 0.3 mg/mL and (**b**) 0.6 mg/mL. Insets, measured resistance change with applied pressure.

**Figure 12:**
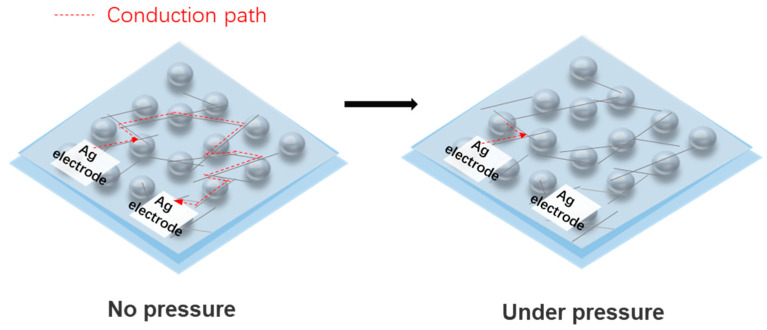
Schematic diagram of the distribution of a low-density AgNWs network on PDMS-expandable microsphere structure with and without applied pressure.

We tested devices fabricated with two different concentrations of the AgNWs ethanol solution, 0.3 mg/mL and 0.6 mg/mL. The results are shown in Figure 11a,b, respectively. The two insets show the resistance change in the two sensors with different applied pressures. It should be mentioned that for the device fabricated with a lower concentration of AgNWs, the resistance became too large to measure when the applied pressure increased to be over 60 kPa. This may be due to the broken of AgNWs network connected at a low density, which results in the shutdown of the conduction path between two Ag electrodes. The sensitivity of the device, S, is calculated as $S=\frac{\delta \left(\frac{\Delta R}{R}\right)}{\delta P}$, where P refers to the applied pressure. Clearly, these two curves in Figure 11a,b can both be divided into two regions according to different sensitivities after linear fitting. For the device fabricated with a lower concentration of AgNWs, sensitivity of 13.93 kPa^−^^1^ and 94.12 kPa^−^^1^ were achieved between 10–40 kPa and 50–60 kPa, respectively. While for the device fabricated with a higher concentration of AgNWs, the sensitivity was 2.99 kPa^−^^1^ and 1.49 kPa^−^^1^ for the pressure range of 20 to 60 kPa and 70 to 120 kPa, respectively. Thus, a lower density of AgNWs networks is preferred to obtain a sensor with high sensitivity in a limited working range, while a higher density of AgNWs networks is desired to achieve a sensor with a large working range but moderate sensitivity. This is because that, as shown in Figure 12, the device with a lower density of AgNWs networks, the conduction paths between two Ag electrodes are very limited; here, only one path is formed in the schematic diagram, and the resistance measured between the two electrodes can be changed greatly under pressure due to significantly reduced number of conduction paths, which results in a high sensitivity but with a limited working range. While for a device with a higher density of AgNWs networks, the original conduction paths are abundant, and shutdown of several conduction paths under pressure has a very small effect on the resistance between the two electrodes, which leads to a lower sensitivity of the sensor, but it can work in a large pressure range. So, by choosing a proper concentration of AgNWs solution, we can design a pressure sensor with high sensitivity, a large working range, or a compromise between these two characteristics according to the application requirements.

### 3.3. Crosstalk between Different Sensors in the Integrated System

**Figure 13:**
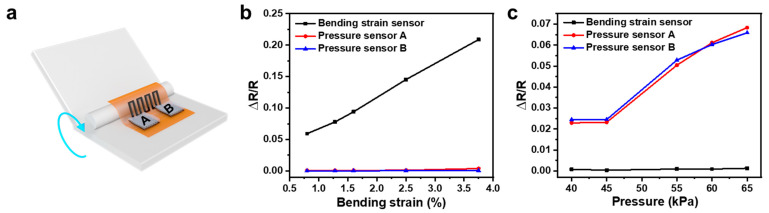
(**a**) Schematic diagram of the experimental setup for the system’s crosstalk characterization. Responses of different sensors in the integrated system under (**b**) a bending strain and (**c**) pressure.

For the integrated system, we first checked the crosstalk between different sensors. The experimental setup is sketched in Figure 13a. When the bending strain was introduced into the system, a linear increase in the relative resistance with strain was observed in the bending strain sensor, while there was almost no response in both pressure sensors A and B, as shown in Figure 13b. Meanwhile, when there was only pressure applied, all the responses happened in the pressure sensors, while the resistance in the bending strain sensor was almost unchanged, as shown in Figure 13c.

### 3.4. Demonstration of Motion Monitoring

**Figure 14:**
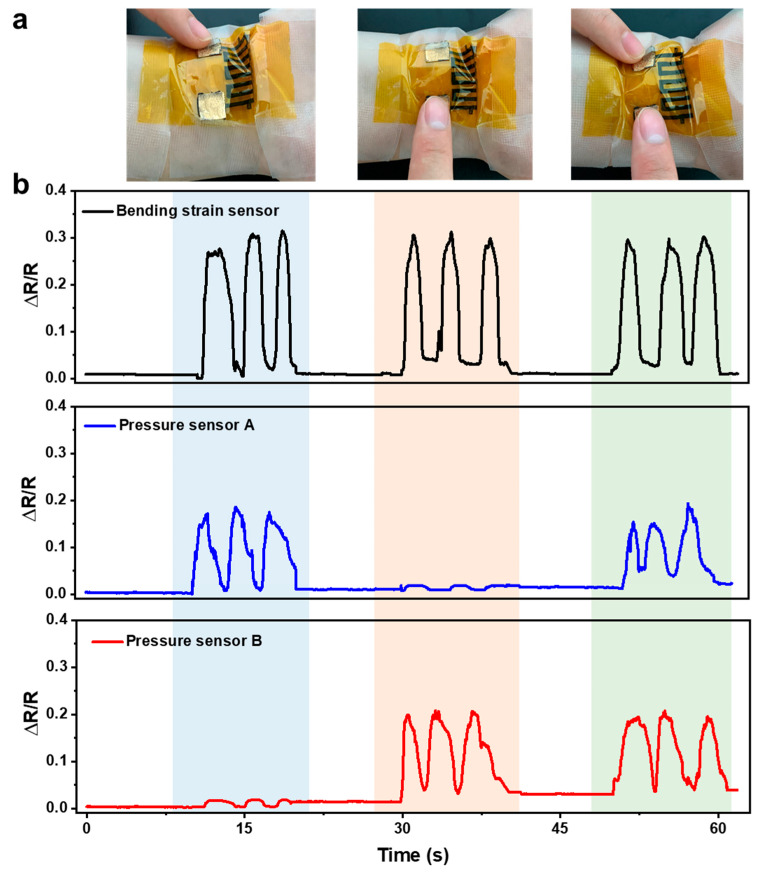
(**a**) Photos showing different actions were carried out when an integrated system was attached to the wrist. (**b**) Signals recorded from different sensors during different actions.

Finally, the integrated system was attached to a person’s wrist for motion monitoring, as shown in Figure 14a. Figure 13b shows the recorded signals from different sensors during different actions. First, the wrist was bent three times, and pressure sensor A was also pressed three times at the same time. As highlighted by the blue strip, the bending strain sensor and pressure sensor A showed three responses correspondingly, while there was no crosstalk signal in pressure sensor B. Then, the wrist was bent three times, and pressure sensor B was pressed, and distinct responses were recorded in the corresponding sensors, as highlighted by the orange strip. Finally, the wrist was bent, and two pressure sensors were pressed together three times. All the sensors showed proper responses (highlighted by the green strip). During these actions, almost no crosstalk was observed between different sensors, and the discrimination of different actions by the integrated system is pretty good.

## 4. Conclusions

In summary, we presented a flexible integrated system for independent bending strain and pressure measurement. The bending strain sensor was constructed with laser-induced graphite, while the pressure sensor was based on a composite structure of PDMS-expandable microsphere and AgNWs. Decent performance was achieved in the integrated system via a simple and low-cost fabrication approach. When the system was attached to the wrist, the independent response to bending strain and pressure enabled the discrimination and monitoring of different mechanical actions on the wrist, showing its great application potentials in smart wearable devices for human–machine interaction.

## Data Availability

The data presented in this study are available on request from the corresponding author.
